# Hypothalamic Obesity following Craniopharyngioma Surgery: Results of a Pilot Trial of Combined Diazoxide and Metformin Therapy

**DOI:** 10.1155/2011/417949

**Published:** 2011-03-01

**Authors:** JillK Hamilton, LouiseS Conwell, Catriona Syme, Alexandra Ahmet, Allison Jeffery, Denis Daneman

**Affiliations:** 1 Division of Endocrinology, Department of Pediatrics, The Hospital for Sick Children, University of Toronto, Toronto, ON, Canada, M5G 1X8; 2 Department of Endocrinology & Diabetes, The Royal Children's Hospital, The University of Queensland, Brisbane, QLD 4072, Australia; 3 Division of Endocrinology, Children's Hospital of Eastern Ontario, Ottawa, ON, Canada, K1H 8L1

## Abstract

*Objective*. To assess the effect of combined diazoxide-metformin therapy in obese adolescents treated for craniopharyngioma. *Design*. A prospective open-label 6-month pilot treatment trial in 9 obese subjects with craniopharyngioma. Diazoxide (2 mg/kg divided b.i.d., maximum 200 mg/day) and metformin (1000 mg b.i.d.). Whole body insulin sensitivity index (WBISI) and area-under-the-curve insulin (AUC_ins_) were calculated. *Results*. Seven subjects completed: 4M/3F, mean ± SD age  years, weight  kg, BMI  kg/m^2^, and BMI SDS . Two were withdrawn due to vomiting and peripheral edema. Of participants completing the study, the mean ± SD weight gain, BMI, and BMI SDS during the 6 months were reduced compared to the 6 months prestudy ( versus  kg, ;  versus  kg/m^2^, ;  versus , , resp.). AUC_ins_ correlated with weight loss (, ) and BMI decrease (, ). *Conclusion*. Combined diazoxide-metformin therapy was associated with reduced weight gain in patients with hypothalamic obesity. AUC_ins_ at study commencement predicted effectiveness of the treatment.

## 1. Introduction

Craniopharyngiomas constitute 9% of childhood intracranial tumors. They are histologically benign, arising from embryonal remnants in the sellar region [1, 2]. In addition to endocrine deficiencies, obesity is common following surgical resection of the tumor, with a significant increase in long-term cardio- and cerebrovascular mortality observed [3–5]. Data from our group, in 17 obese youth with craniopharyngioma, show significantly more features of the metabolic syndrome (66% versus 15%), higher acute insulin secretion in response to an oral glucose tolerance test (OGTT), and a higher prevalence of glucose intolerance (40% versus 0%) compared to age- and BMI-matched controls [6]. 

The mechanisms contributing to hypothalamic obesity following surgery for craniopharyngioma, likely include one or both of the following: (i) damage to hypothalamic nuclei and neuronal circuits involved in appetite and body weight regulation and (ii) a primary defect of insulin hypersecretion due to hypothalamic damage-induced vagal efferent stimulation with increased weight gain and compensatory insulin resistance [7, 8].

Given the serious challenges posed by the ongoing weight gain and the lack of effective interventions, we developed a novel therapeutic approach with combination therapy aimed both at decreasing insulin secretion using diazoxide and simultaneously enhancing insulin action through the use of metformin.

Neither metformin nor diazoxide the combination has been tested previously in children with hypothalamic obesity due to intracranial damage. The oral route of medication administration, safety profile, decreased cost, and their mechanisms of action support their use in combination in this situation. We hypothesized that the combined metformin and diazoxide therapy would lower insulin secretion while minimizing the risk of hyperglycemia, leading to weight loss and improvement in metabolic status. 

## 2. Materials and Methods

An open-label, 6-month duration, pilot treatment trial of combined diazoxide-metformin treatment was employed in children with obesity following craniopharyngioma surgery. 

Forty-six children under the age of 19 years treated surgically for craniopharyngioma tumour are currently being followed at the Hospital for Sick Children, Toronto. Approximately 50% are obese defined by a BMI ≥ 95th centile for age and gender assessed from the updated Centre for Disease Control growth charts [9]. All of these children have hypopituitarism requiring hormone replacement therapy. They are assessed regularly in our comprehensive care clinic for children with tumour-related hypothalamic obesity. This is a multidisciplinary care setting staffed by an endocrinologist, dietitian, exercise consultant, and psychologist with expertise in treatment of obesity. Patients may attend monthly to receive support in regard to lifestyle counseling.

Subjects who met the following inclusion criteria were recruited into the study: 

(i)history of a craniopharyngioma in the sellar/suprasellar region with hypothalamic involvement at least one year following surgery; 

(ii)stable hormone replacement therapy for more than one year (with one or more of thyroxine, hydrocortisone, desmopressin, sex steroids, and growth hormone); 

(iii)obesity, defined as BMI > SDS for age and gender, or those with BMI < 85th percentile before tumor diagnosis but weight gain >2SD above mean for age for 1 year following tumor treatment. These criteria were chosen based on studies in this population showing favourable changes using a long-acting octreotide analogue [10];

(iv)age 9–19 years; 

(v)minimum of 6 months of standard diet and exercise intervention in our existing comprehensive care clinic (run-in period). During this time, individuals met with each of the interdisciplinary team members at the monthly clinic to learn about increased physical activity, healthy dietary intake and receive psychologic support to promote change in behavior. This time frame was chosen to allow a period of prospective measurements to establish individual baseline slope of change in BMI SDS prior to initiation of active treatment with diazoxide and metformin. 

Exclusion criteria included the following. 

(i)Contraindications for metformin or diazoxide use (history or evidence of cardiac, renal, or progressive hepatic disease, diabetes, or hypoxic conditions). 

(ii)Pharmacologic doses of glucocorticoids or use of other medications known to affect glucose metabolism (e.g., beta-blockers, oral hypoglycemics) within 6 months of study onset.

(iii)Growth hormone (GH) initiation in the preceding 6 months or planned initiation in the next 6 months (to rule out potential confounding effect of GH on weight/body composition and glucose metabolism).

(iv)Use of other weight loss medications.

(v)Inability of the family and/or patient to comply with study protocol. 

(vi)Non-English speaking.

## 3. Treatment Protocol

Following a 6-month period of diet and exercise counseling, subjects received diazoxide (2 mg/kg divided b.i.d., maximum 200 mg/day divided twice daily) and metformin (2000 mg per day divided twice daily) given with meals for 6 months. This dose of diazoxide was chosen because it fits within the pediatric dosing guidelines to treat children with hyperinsulinism (3–8 mg/kg/day) and is similar to that used in previous studies in obese adults (100 mg b.i.d-t.i.d.) [11].

Diazoxide was initiated at half the dose (100 mg per day) for 2 weeks to allow the insulin sensitizing action of metformin to take effect. Metformin was initiated at 500 mg twice daily and increased to 1000 mg twice daily over a one-week period to minimize gastrointestinal side effects. This dose is typically used in adolescents and adults with polycystic ovarian disease or type 2 diabetes [12].

## 4. Baseline and Posttreatment Assessment

### 4.1. History and Physical Exam

Family and medical history was obtained from the hospital chart during clinic review and from the patient. Information obtained included duration since surgery for the tumor, endocrine sequelae, family history of metabolic syndrome (hypertension, dyslipidemia, cardiovascular disease, type 2 diabetes), and current medications. Physical examination included assessment of systolic and diastolic blood pressure recorded as the mean of 3 readings taken 1 minute apart in the right arm in the seated position using a Dinamap device and appropriately sized cuff. Puberty was assessed using the staging method of Tanner [13, 14]. A standard, calibrated scale and wall-mounted stadiometer were used to measure weight and height, and BMI was calculated as wt (kg)/ht (m)^2^. BMI standard deviation scores (SDS) were calculated based on data from the Center Disease Control (http://www.cdc.gov/nccdphp/dnpa/growthcharts/resources/sas.htm). 

### 4.2. Glucose and Insulin Dynamics

Following an overnight fast, subjects arrived at the Clinical Investigation Unit at 8 : 00 AM for an OGTT (1.75 mg/kg, max 75 g), and venous blood samples were obtained at 0, 30, 60, 90, and 120 minutes to screen for impaired glucose tolerance and type 2 diabetes [15]. Using values derived from the OGTT, whole body insulin sensitivity index (WBISI) was calculated using the Matsuda index calculated as 10,000/*√*((fasting glucose × fasting insulin) × (mean glucose × mean insulin)), where glucose is measured in mmol/L and insulin in pmol/L. This has been validated against gold-standard measures of insulin sensitivity in adults and in children [16, 17]. The area under the curve of insulin (AUC_ins_) during OGTT was calculated as an index of insulin secretion using the trapezoid method [18].

Insulin was measured by chemiluminescence using the Siemens Immulite 2500 (range of assay 15–2165 pmol/L, intra- and interassay coefficient of variation (CV) <7.6%). Leptin was measured using enzyme-linked immunosorbent assay supplied by Diagnostic Systems Laboratories Inc. (range 0.1–50.0 ng/mL; interassay CV 1.5–6.2%). Adiponectin was measured by ELISA (Linco Research Inc., range: 1–100 ng/mL; interassay CV 2.4–8.4%). Adiponectin and leptin are synthesized and secreted from adipose tissue and are correlated negatively and positively, respectively, to insulin resistance and obesity in children and adolescents [19–22]. Fasting triglycerides (TG), total cholesterol, LDL, high-density lipoprotein (HDL) cholesterol, and apolipoproteins were measured by standard enzymatic methods. Low-density lipoprotein (LDL) cholesterol was calculated using the following formula: LDL cholesterol = total cholesterol − TG/2.2 + HDL cholesterol.

## 5. Monitoring

Subjects were assessed monthly for dietary and exercise consultation receiving ongoing support similar to the 6-month run-in period and monitoring of side effects of medication. Subjects were also contacted by phone weekly for the first month to review potential side effects and were taught to perform urine dipstick to assess for glucosuria. Development of diabetes or significant medication side effects were considered reasons for withdrawal. Compliance was assessed at monthly visits by tablet count. 

This study was approved by the Research Ethics Board at the Hospital for Sick Children, Health Canada Clinical Trials Application (http://clinicaltrials.gov/, identifier: NCT00892073). Informed consent was obtained from both parents and subjects prior to study commencement. A Data Safety Monitoring Committee (DSMC) received monthly reports of recruitment, laboratory results, and any adverse events and met every 3 months during the study duration. They also received immediate notification of any serious adverse events.

## 6. Statistical Analysis and Power Calculation

Statistical analysis was performed using SAS software (SAS version 8.2, Cary, NC, 1999). Continuous variables were expressed as mean and standard deviation. Comparisons of weight parameter changes, biochemical tests, and calculated indices of insulin secretion and sensitivity in the 6 months prior to study entry and during the 6 months on study medication were compared using the paired -test with Bonferroni correction for multiple -tests. Pearson's correlation analysis was performed between measures of changes in weight parameters and biochemical parameters at study onset (AUC_ins_, WBISI, leptin, adiponectin). Log transformations were performed on nonnormative variables for analyses and back-transformed for data presentation. 

For this pilot study, it was estimated that recruitment of 8 subjects should be adequate to achieve 80% power to detect a difference of change in BMI of 2.1 kg/m^2^ between the 6-month run-in period and the 6-month treatment period based on a pilot study of octreotide in a similar population [8].

## 7. Results

Nine (4 female) subjects were eligible and all consented to participate. All had evidence of hypothalamic damage on MRI following surgical treatment for craniopharyngioma. Two subjects did not complete the study secondary to adverse medication effects. One female subject was withdrawn after 2 months of treatment due to the development of pedal edema. A second female participant was withdrawn after 4 months of treatment due to mildly elevated hepatic enzyme levels (ALT 120; AST 134 U/L) and vomiting. These adverse events resolved after medication cessation. Blood pressure and glucose tolerance remained normal in all of the participating subjects.

Considering the seven subjects completing the study, the mean ± SD age was  years, time since diagnosis  years, and Tanner stage . The mean ± SD weight was  kg, BMI  kg/m^2^, and BMI SDS . All participants had panhypopituitarism, requiring hormone replacement, and were receiving levo-thyroxine, hydrocortisone, and desmopressin replacement. Seven were receiving growth hormone (mean  years), and 8 were receiving sex steroid replacement. All had normal glucose levels both fasting and 2 hours following an OGTT. Patient characteristics at baseline are provided in Table 1, and biochemical tests before and after study are shown in Table 2.

**Table 1:** Patient characteristics at baseline.

**Subject**	**Age (years)**	**Sex**	**Years since Dx**	**Weight (kg)**	**BMI (kg/m^2^)**	**BMI SDS**
1	15.8	F	4.4	95.5	34.7	2.03
2	18.3	F	4.7	135.3	45.4	2.41
3	16.2	F	6.3	92.4	35.4	2.00
4*	11.4	F	5.7	66.0	32.8	2.32
5	16.1	M	8.0	121.6	37.7	2.54
6*	15.9	F	8.7	156.5	65.1	2.96
7	14.7	M	7.3	75.7	28.3	1.8
8	17.1	M	8.1	112.0	34.0	2.14
9	9.2	F	2.8	56.8	29.6	2.48

Dx: diagnosis of craniopharyngioma; BMI: body mass index; SDS: standard deviation score. *Denotes subjects who were withdrawn from the study due to vomiting with mild elevation of hepatic enzymes (subject 4) and peripheral edema (subject 6).

**Table 2:** Biochemical measures at baseline and after 6 months of treatment (mean ± SD).

**Test**	**Initial**	**Final**
Fasting TG (mmol/L)	1.97 ± 1.05	1.96 ± 0.63
HDL (mmol/L)	1.00 ± 0.30	0.93 ± 0.41
LDL (mmol/L)	2.18 ± 0.94	2.43 ± 0.91
Total cholesterol (mmol/L)	4.07 ± 0.81	4.26 ± 0.90
WBISI	2.2 ± 1.1	5.7 ± 8.1
AUC_ins_ (pmol/L)	2078.8 ± 668.8	1438.3 ± 988.5
Adiponectin (mg/mL)	10.01 ± 4.39	8.17 ± 3.46
Leptin (ng/mL)	27.64 ± 22.14	23.55 ± 25.34
HbA1c (%)	5.0 ± 0.3	4.8 ± 0.5
AST (U/L)	31.0 ± 6.95	30.4 ± 16.80
ALT (U/L)	28.3 ± 7.45	40.4 ±48.79
Fasting glucose (mmol/L)	4.4 ± 3.60	4.7 ± 0.69
Fasting insulin (pmol/L)	174.3 ± 87.79	130.2 ± 99.56
2 hour glucose (mmol/L)	5.4 ± 0.96	6.2 ± 1.36
2 hour insulin (pmol/L)	995.3 ± 531.19	735.0 ± 683.97

*P* value: not significant for all variables shown above. TG: triglycerides; HDL: high-density lipoprotein cholesterol; LDL: low-density lipoprotein cholesterol; WBISI: whole body insulin sensitivity index; AUC_ins_: area-under-the-curve insulin; HbA1c: hemoglobin A1c; AST: aspartate aminotransferase; ALT: alanine aminotransferase.

**Figure 1:**
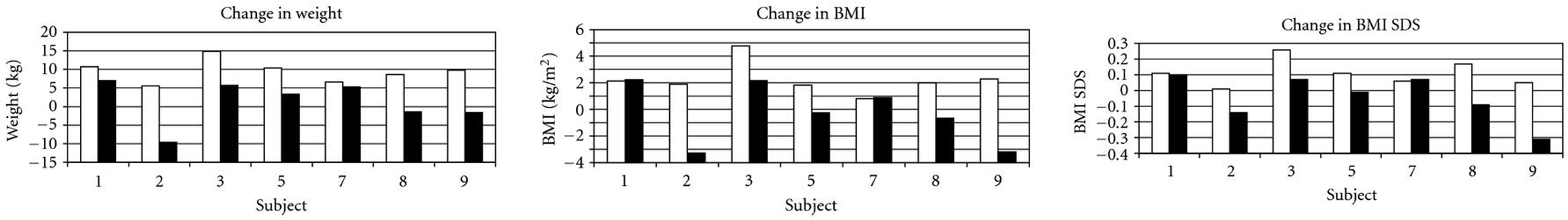
Changes in individual (a) weight, (b) BMI, and (c) BMI SDS during the 6 months before (light bars) and after 6 months (dark bars) of metformin-diazoxide treatment.

Of participants completing the study, the mean ± SD weight gain during the 6 months of the study was significantly reduced compared to that in the 6 months prestudy ( versus  kg, ) (Figure 1(a)). The mean BMI ( versus  kg/m^2^, ) was also significantly reduced during the study period (Figure 1(b)). The change in BMI SDS was also significantly reduced in the 6 months during the study period ( versus , ) (Figure 1(c)).

**Figure 2:**
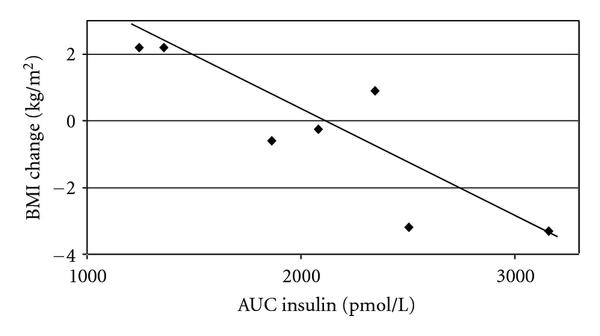
BMI Change during 6 months treatment with diazoxide and metformin relative to AUC_ins_ at the beginning of Treatment (, ).

**Figure 3:**
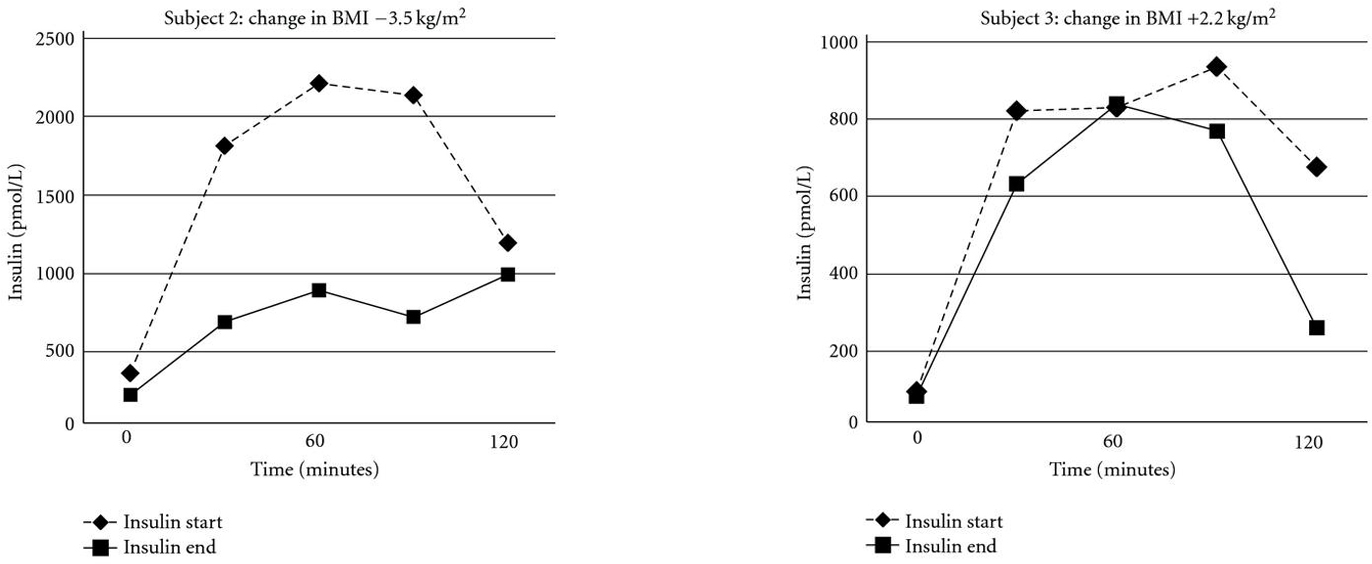
Insulin secretion before and after treatment and changes in BMI in a responder (a) and a nonresponder (b).

AUC_ins_ at the commencement of the study was correlated with weight loss (, ) and BMI decrease (, ) during the study (Figure 2). The positive correlation between WBISI and BMI decrease approached statistical significance (, ) while that with weight did not (, ). There was no significant correlation between any of the weight parameters and leptin or adiponectin at the start of the study (data not shown). Figure 3 provides an example of the pre- and posttreatment insulin curves in a "responder" and a "nonresponder." 

## 8. Discussion

In this study, a novel therapeutic approach based on combined treatment both to decrease insulin secretion using diazoxide and at the same time enhancing insulin action through the use of metformin was utilized to treat children with hypothalamic obesity secondary to craniopharyngioma surgery. Overall, our subjects exhibited a decrease in weight gain and BMI during the 6-month treatment period, although there was some variability in response to treatment. Three of seven subjects lost weight during the treatment period; however, all exhibited less weight gain than in the preceding 6 months. Most interestingly, subjects with the highest insulin secretion at the start of the study experienced the greatest weight loss response to therapy.

Two synergistic mechanisms likely contribute to the hypothalamic obesity following surgery for craniopharyngioma, although their relative contributions are not known: (i) damage to hypothalamic nuclei and neuronal circuits involved in appetite and body weight regulation and (ii) a primary defect of insulin hypersecretion due to hypothalamic damage-induced vagal efferent stimulation with increased weight gain and compensatory insulin resistance [7, 8]. Damage to critical areas involved in hunger, satiety, and energy homeostasis impairs signaling and feedback mechanisms. Experimental rat models, following damage to the ventromedial (VMH) hypothalamus, demonstrate hyperphagia, obesity, hyperinsulinism, and insulin resistance [23]. Furthermore, pancreatic vagotomy in these animals results in weight loss, implicating the role of insulin hypersecretion in their weight gain [7]. There is a case report of a woman with hypothalamic obesity who responded to truncal vagotomy with significant weight loss [24]. Enhanced vagal efferent stimulation leading to exaggerated insulin release may occur via several mechanisms. These include (i) a "cephalic" or premeal vagal stimulation of muscarinic receptors linked to sodium channel in the pancreatic *β*-cell membrane resulting in slight depolarization and "priming" of the *β* cell for glucose-mediated depolarization, (ii) increased production of *β*-cell phospholipases important for downstream mediators of intracellular and vesicular release of calcium and insulin, and (iii) stimulation of the insulin secretagogue glucagon-like peptide-1 (GLP-1) from intestinal L cells [25]. There is also some suggestions that decreased sympathetic tone may also contribute to autonomic dysregulation and hypothalamic obesity [26]. A recent pilot study of caffeine and ephedrine given to 3 patients with hypothalamic obesity resulted in a mean weight loss of 14% with 2 of the 3 patients sustaining weight loss for at least 2 years [27]. 

In this study, diazoxide was chosen as the therapeutic agent as it effectively decreases insulin release by binding to the KATP channel and preventing closure [11]. Oral diazoxide is used primarily in the treatment of neonatal and childhood hyperinsulinemic hypoglycemia at doses of 3–8 mg/kg/day [11]. Diazoxide has been evaluated in one study in 24 obese hyperinsulinemic adults to promote weight loss at doses of 2 mg/kg per day (up to 200 mg daily) for 8 weeks. Treatment resulted in a greater weight loss (% versus %, ), greater decrease in body fat ( kg versus  kg, ), and greater attenuation of acute insulin response to glucose compared to a low calorie diet alone. It was well tolerated, without glucose intolerance [28]. Octreotide, another agent used in the treatment of hyperinsulinemic hypoglycemia, has been studied as a weight loss agent in the context of hypothalamic obesity. In a 6-month pilot octreotide treatment trial in 8 obese children with hypothalamic obesity, children exhibited a weight loss of  kg () and a decrease in BMI ( kg/m^2^, ). The degree of weight loss correlated to the change in insulin response [8]. A randomized controlled trial of long-acting octreotide by the same group was performed in 172 obese adults with insulin hypersecretion, defined by OGTT. Weight loss during this study was modest, approximately 2%, with a corresponding decrease in BMI of approximately 0.75 kg/m^2^. As in the hypothalamic obesity study with octreotide in children, those with higher insulin secretion at study start gained the most benefit in terms of weight loss [10].

One of the significant concerns of the use of diazoxide and other agents to decrease insulin secretion is the potential to lead to significant side effects including gastrointestinal disturbances, biliary gallstones, edema, and hyperglycemia. In our study, 2 subjects were withdrawn, one for significant gastrointestinal symptoms and the second due to the development of peripheral edema. We did not experience any subjects developing hyperglycemia, and this may have been due to the addition of metformin. 

Metformin is an oral antihyperglycemic agent which acts predominantly to decrease hepatic gluconeogenesis and has a modest effect on peripheral glucose uptake into insulin-sensitive tissues. In adults with and without diabetes, metformin has been associated with weight loss or a decreased rate of weight gain and body fat accumulation [29]. This can be attributed to both a net decrease in caloric intake due to appetite suppression and beneficial effects from improvement of insulin and glucose dynamics. Four randomized placebo-controlled trials with treatment of 1000 to 1700 mg divided twice daily, of 2–6 month duration in obese (BMI > 30 kg/m^2^) adolescents, showed modest reduction in BMI, serum leptin, glucose, and insulin levels and an increase in insulin sensitivity [29–32]. The largest of these studies conducted over a 6-month period in 120 obese adolescents demonstrated a statistically significant decline in body mass index (from  to  kg/m^2^, ) [30]. 

Our study is limited by the small number of participants and lack of randomization. It is possible that the most motivated individuals chose to participate in the study and that their improvement was related to ongoing lifestyle changes rather than medication per se. However, the facts that insulin decreased in those who lost most weight and increased AUC insulin prior to treatment predicted improved response to medication suggest that mechanisms related to therapy (in particular, diazoxide) led to favourable changes in weight. It is not possible from this study to dissect out fully, whether diazoxide or metformin alone would have led to similar results. Further studies in this population will be required.

In children with hypothalamic obesity with demonstrated inexorable weight gain and who are at very high risk for cardiometabolic complications, we found that combination treatment with diazoxide and metformin resulted in less weight gain and favourable changes in BMI. Although the medication was generally well tolerated, two subjects were withdrawn, one due to side effects of diazoxide (edema) and the second to adverse gastrointestinal symptoms secondary to metformin. This highlights the need for careful monitoring when using these medications. The outcome of this pilot study is encouraging and does provide necessary data for a larger randomized trial in individuals with hypothalamic obesity including children with craniopharyngioma and others who exhibit hypersecretion of insulin. If also positive, this would lead to extrapolation of this approach to other obese, hyperinsulinemic groups. 
